# A Solvent-Free Approach for Converting Cellulose Waste into Volatile Organic Compounds with Endophytic Fungi

**DOI:** 10.3390/jof4030102

**Published:** 2018-08-26

**Authors:** Tyler Maxwell, Richard G. Blair, Yuemin Wang, Andrew H. Kettring, Sean D. Moore, Matthew Rex, James K. Harper

**Affiliations:** 1 Department of Chemistry, University of Central Florida, 4111 Libra Drive, Orlando, FL 32816, USA; Tyler.Maxwell@knights.ucf.edu (T.M.); Yueminwang@knights.ucfe.edu (Y.W.); Matthew.Rex@ucf.edu (M.R.); 2 Florida Space Institute, University of Central Florida, 12354 Research Parkway, Suite 214, Orlando, FL 32826, USA; Richard.Blair@ucf.edu; 3 Burnett School of Biomedical Sciences, University of Central Florida, 4110 Libra Dr., Orlando, FL 32816, USA; Akettring@knights.ucf.edu (A.H.K.); Sean.Moore@ucf.edu (S.D.M.)

**Keywords:** endophytic fungi, Mechanocatalysis, cellulose degradation, volatile organic compounds, myco-diesel, *Hypoxylon*

## Abstract

Simple sugars produced from a solvent-free mechanocatalytic degradation of cellulose were evaluated for suitability as a growth medium carbon source for fungi that produce volatile organic compounds. An endophytic *Hypoxylon* sp. (CI-4) known to produce volatiles having potential value as fuels was initially evaluated. The growth was obtained on a medium containing the degraded cellulose as the sole carbon source, and the volatile compounds produced were largely the same as those produced from a conventional dextrose/starch diet. A second *Hypoxylon* sp. (BS15) was also characterized and shown to be phylogenetically divergent from any other named species. The degraded cellulose medium supported the growth of BS15, and approximately the same quantity of the volatile compounds was produced as from conventional diets. Although the major products from BS15 grown on the degraded cellulose were identical to those from dextrose, the minor products differed. Neither CI-4 or BS15 exhibited growth on cellulose that had not been degraded. The extraction of volatiles from the growth media was achieved using solid-phase extraction in order to reduce the solvent waste and more efficiently retain compounds having low vapor pressures. A comparison to more conventional liquid–liquid extraction demonstrated that, for CI-4, both methods gave similar results. The solid-phase extraction of BS15 retained a significantly larger variety of the volatile compounds than did the liquid–liquid extraction. These advances position the coupling of solvent-free cellulose conversion and endophyte metabolism as a viable strategy for the production of important hydrocarbons.

## 1. Introduction

The endophytic fungi are organisms that colonize the tissue of living plants. In most cases, this relationship is asymptomatic and may even provide benefits to plants [1]. Endophytes have been studied extensively and found to produce a remarkable variety of natural chemical products [2]. While much of the interest has focused on bioactive compounds, the production of other important compounds has also been reported. A recent noteworthy discovery is that certain endophytes can produce hydrocarbons that have the potential to be used as fuels or fuel additives [3]. These products have been compared to diesel fuel and even described as “myco-diesel”, because they include compounds normally associated with diesel fuel. Over the past decade, interest in fungi producing volatile organic products with the potential for use as fuels has increased, and several studies have identified potentially useful fungi [4,5,6,7,8,9,10]. Related work has also identified fungi producing volatile products but has not focused on their potential usefulness as fuels [11,12,13,14,15,16,17,18,19,20,21,22,23,24,25].

The availability of hydrocarbon fuels from fungi complements fuel products produced by other organisms. For example, certain algae produce aliphatic fatty acids and considerable effort has been expended into developing these into viable biofuels [26]. Likewise, yeast fermentation has been prominently utilized to convert carbohydrates from corn into ethanol for fuel [27]. In general, fungal products contain a more complex variety of volatile compounds than either algae or yeast, including ketones, esters, alcohols, and a remarkable variety of hydrocarbon products. All of these biofuels complement more conventional fuels and thus represent important pathways worthy of exploration given the current interest in developing alternative fuels. However, one of the concerns that exists when producing hydrocarbon fuel from fungi is that they require a refined carbohydrate source (e.g., sucrose) in their diet. There has been debate regarding the suitability of devoting carbohydrates to fuel production. A solution to this dilemma would be to find an alternative food source for the fungi.

Recently, a “green chemistry” mechanocatalytic method has been reported that allows cellulose waste products to be converted into simple carbohydrates [28]. This process involves ball milling performed in the solid state and is thus entirely solvent free and capable of rapidly producing large quantities of carbohydrates (see experimental). The major water-soluble products from this process have been shown to be glucose, fructose, and levoglucosan. No oligosaccharides larger than dimers survive the milling, even after short processing times (e.g., 30 min) [28]. This process has been successfully demonstrated using a remarkable variety of cellulose-based feedstock materials from plants (Table 1) and includes numerous materials normally regarded as unusable waste such as orange peels, cherry pits, coffee grounds, and discarded newspaper.

At the present time, however, it has not been demonstrated that fungi can actually grow on the carbohydrates created from the cellulose breakdown process. The aim of this manuscript is to demonstrate that carbohydrates produced from this solvent-free degradation process are a suitable carbon source for fungal growth and that the volatile products produced from the degraded cellulose closely match compounds produced from a more conventional diet. In the following, sugars from only one cellulose source (oak) are tested. The results from other materials in Table 1 are expected to give similar results, as it has been demonstrated that cellulose from various sources consistently breaks down into simple sugars [28].

## 2. Materials and Methods

The cellulose employed in this study to create the simple sugars was obtained from water oak (*Quercus nigra*) sawdust sourced from a local sawmill. The oak was dried at room temperature to a moisture content of <10% and cut into 2 cm or smaller pieces. Delaminated kaolinite (Kaopaque 10, IMERYS) was used as received.

The mechanical processing of cellulose employed 8000M and 8000D mixer mills (SPEX Certiprep, Metuchen, NJ, USA). Two grams of a 1:1 mixture of the kaolinite clay catalyst and biomass source were processed for two hours in 65-mL vials (1.5′′ ID × 2.25′′ deep) made of 440C steel, utilizing three 0.5-inch diameter balls composed of the same material as the milling vial. Energy was applied in 30-min intervals with 30 min of cooling time to minimize the effects of frictional heating. Hydrolysis of hemicellulose and cellulose (holocellulose) was monitored gravimetrically. Conversion of holocellulose to water-soluble oligosaccharides was determined by stirring 0.1 g of the reaction mixture in 30 mL of water. The production of water-soluble products was measured by filtration through a 47-mm diameter Whatman Nuclepore^®^ track etched polycarbonate membrane filter with a pore size of 0.220 µm. The residue was dried in a 60 °C oven for 12 h and then weighed.

The potato dextrose broth and agar were purchased from Becton Dickinson. Ammonium sulfate, acetonitrile, ethyl acetate, anhydrous magnesium sulfate, and methanol were purchased from Fisher Scientific. A sample of 1,8-cineole was obtained from TCI chemicals (Portland, OR, USA). The yeast nitrogen base was purchased from Sigma Aldrich. HyperSep C-18 solid-phase extraction columns (1 g bed weight) were purchased from Thermo Scientific. Potato dextrose agar was purchased from Microtech Scientific. All the reagents were used as received.

The isolation of the *Hypoxylon* sp., BS15, was from branch clippings of a *Taxodium distichum* (Bald Cyprus) gathered near Orange City, Florida, USA. The branches were treated with 70% ethanol, flame sterilized, and then dried in a sterile laminar-flow hood. A sterile knife blade was then used to cut away the outer tissue from the clipping, and a square wedge of the inner tissue was placed on water agar. This dish was incubated, and any fungal hyphae observed growing from the sample were transferred onto separate plates of potato dextrose agar.

The potato dextrose broth was prepared by adding 2.4 g of the potato dextrose broth to 100 mL of purified water in a 500-mL Erlenmeyer flask. The flask was then sealed with aluminum foil and autoclaved for 15 min for sterilization. The fungi of interest (CI-4 or BS15) were then added to the sterile broth, and it was resealed with foil. Cellulose broth was prepared using 250 mL of purified water, 5 g of degraded cellulose, 1.5 g of ammonia sulfate, and 1.7 g of the yeast nitrogen base without amino acids. In both growth media, the fungi were then left to grow for 25 days in the lab at 20–25 °C without stirring. Each broth was then vacuum filtered twice through Whatman Grade 4 filter paper to remove all particulates.

A control sample containing cellulose not subjected to the mechanocatalytic degradation process was prepared by adding 2 g of finely ground cellulose powder and 0.5 g of ammonium sulfate to 250 mL of distilled water. This medium was autoclaved for 15 min, and, after cooling, two separate solutions were prepared by adding CI-4 or BS15 to the liquid. This culture was allowed to grow for 2 weeks at 20–25 °C without stirring.

For the solid-phase extraction of the fungal volatile compounds, a C-18 cartridge was first washed with 4 mL of methanol and then with 4 mL of water. Filtered fungal broth (50 mL) was then passed through the column slowly under vacuum. The column was washed again with 4 mL of water to remove any contaminants and then dried by drawing air through the column for 15 min. The retained compounds were then eluted by passing acetonitrile through the column. A clear brown solution was typically recovered from this process. The eluent was then filtered with a 0.22-µm syringe filter prior to analysis.

For the liquid–liquid extractions, a total of 300 mL of the filtered fungal broth was shaken in a separatory funnel with 50 mL of ethyl acetate. The ethyl acetate was then separated from the water and dried over anhydrous magnesium sulfate. The solution was then filtered with a 0.22-µm syringe filter prior to analysis.

The gas chromatography/mass spectrometry (GC/MS) analysis for the volatile compounds was performed using a method described previously with slight modification [29,30]. An Agilent 6850 was used with a 5975CVC MS detector and a Restek Rxi-5HT capillary column (30 m × 0.25 mm, film thickness 0.25 µm). The carrier gas was ultrahigh purity helium with a one cm^3^/min constant flow rate and an initial column head pressure of 77 kPa. The injector split was set to 250 °C at a 20:1 split ratio with 1-μL volume per injection. The column oven temperature was programmed to 45 °C with an initial temperature hold for 1 min with a 10 °C/min ramp to 100 °C and hold for 5 min, followed by a 5 °C/min ramp to 200 °C and a hold for 5 min. The detector was set at a constant 280 °C and set to scan 30–350 m/z. The data acquisition and processing were performed on Agilent MSD ChemStation software. The identification of the compounds was made via library comparison using the National Institute of Standards and Technology (NIST, Gaithersburg, MD, USA) database.

For DNA extraction from BS15, a small sample of the fungal tissue (50–100 mg) was collected into a microcentrifuge tube from the surface of a potato dextrose agar plate after 1 week of growth at room temperature. The tissue was lysed using a FastPrep Homogenizer (MP Biomedicals, Santa Ana, CA, USA) by zirconia–silica bead beating in 1 mM of sodium dodecyl sulfate, 5 mM of EDTA, and 10 mM of Tris-HCl, pH 8.0 with 10 µg/mL RNase A. The lysate was centrifuged, and then, the DNA was purified from the supernatant by silica column binding in guanidinium thiocyanate [31].

Diagnostic gene sequences used for identification by genetic barcoding were amplified by a routine polymerase chain reaction (PCR) with Taq [32,33]. Primers ITS1-F_KYO1 and ITS4_KYO1 were used to target the internally transcribed spacers (ITS1 and ITS2) and the flanking portions of the ribosomal RNA encoding genes (SSU, 5.8S, and LSU) [34]. The protein-coding genes α-actin and β-tubulin were amplified by primers ACT-512F/ACT-783R and T1/T22, respectively [35,36]. The PCR products were visualized by agarose gel electrophoresis, similarly purified by silica column binding, and then sequenced commercially (GENEWIZ, Plainfield, NJ, USA). The sequences were deposited in GenBank under accession number MH223406 (ITS), MH465497 (actin), and MH465498 (tubulin).

The ribosomal gene sequences were analyzed with a series of BioPython-based scripts [37]. First, full-length ITS sequences were extracted via ITSx and used to locally query the UNITE+INSDC fungal database by BLAST search [38,39,40]. Based on these search results, relevant taxa were selected, and a list was compiled of all the unambiguous binomial species within these taxa. The corresponding UNITE records were pooled and analyzed by ITSx. For each species, a single representative full-length ITS2 record was chosen for alignment. Relevant α-actin and β-tubulin records used in alignment were retrieved from GenBank [41].

The phylograms were generated using MEGA software [42]. The sequences were aligned by the MUSCLE algorithm and then clustered by the maximum likelihood method with 1000 bootstrap replicates [43,44]. Both the α-actin and β-tubulin sequences were treated as protein-coding during the phylogenetic analyses, while ITS sequences were not. All other settings in MEGA were unchanged and no manual modifications were made during the alignment or clustering. The resultant phylograms were exported and visualized via Interactive Tree of Life (iTOL) web software [45]. The nodes were pruned on the basis of relatedness to BS15 and intra-generic species richness. The alignments and phylograms were deposited in TreeBase under submission number 23089.

## 3. Results and Discussion

### 3.1. Growth of the Hypoxylon CI-4 on Degraded Cellulose

**Figure 1:**
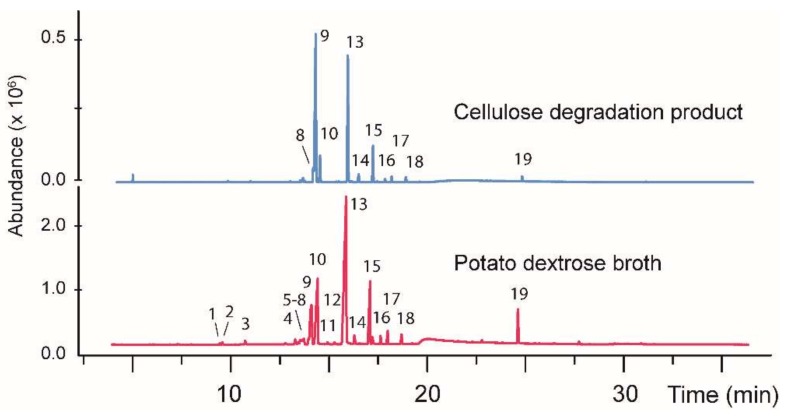
A gas chromatogram showing the volatile organic products produced by the fungus CI-4 growth on a conventional media (**bottom**) versus a diet containing carbohydrates produced from cellulose degradation (**top**). The nominal masses for each numbered peak are given in Table 2. In each case a control sample was also analyzed consisting of the growth medium without fungi added. This solution was processed identically to the fungi-containing samples. In each case, no peaks from the control samples corresponded to any of the peaks shown above.

As an initial test of the feasibility of using carbohydrates from mechanocatalytic cellulose degradation as a fungal diet, a *Hypoxylon* sp. was added to a growth medium consisting of the degraded cellulose as the sole carbon source (see experimental). A control sample was also prepared, having the fungus on a conventional diet of potato dextrose broth. The particular *Hypoxylon* fungus used for this study (designated CI-4) was selected because it has been previously shown to produce a diverse variety of volatile organic hydrocarbons [46,47]. Both cultures exhibited similar fungal growth and were incubated for three weeks. The hydrocarbon fraction was extracted from the growth media using a solid-phase extraction process (see experimental). A gas chromatography/mass spectrometry (GC/MS) analysis exhibited a diverse range of volatile products, as expected from the previous study [46]. A comparison of the volatile compounds produced from each growth condition is illustrated in Figure 1 and demonstrates that the degraded cellulose material produces the same major products as a conventional carbohydrate-rich diet. A notable difference, however, between the growth media is that the amounts of the volatile compounds produced from the cellulose degradation products were roughly two–five times less than the same products produced from the potato dextrose broth.

The molecular masses and tentative identification of individual compounds from CI-4 were made by comparing the mass spectrum of each peak against the data in the NIST database. Although the nominal masses were obtained in all cases, most compounds were not identifiable. All the results are summarized in Table 2. Also included in Table 2 are the results from the analysis of a second fungus (BS15, described below).

To verify that the volatile compounds produced from CI-4 and grown on the degraded cellulose are the result of the presence of simple sugars rather than residual cellulose, a control containing cellulose not degraded by the mechanocatalytic process was also prepared for comparison (see Materials and Methods). After two weeks of incubation on this medium, CI-4 showed no growth.

A notable difference between the compounds extracted here by the solid-phase extraction and the previous study of CI-4 [46,47] is that the solid-phase extraction failed to recover some of the early eluting peaks. As discussed below, a liquid–liquid extraction demonstrated that these compounds are, in fact, present, and the cause of their omission from the solid-phase extraction sample is currently under investigation.

### 3.2. Phylogenetic Characterization of a New Hypoxylon sp., BS15

Recently a second fungus producing volatile organic products was isolated from a Bald Cyprus (*Taxodium distichum*) near Orange City, FL (USA). This fungus, designated BS15, was selected for study based on the serendipitous observation that compounds having a distinctive odor were produced.

The identification of BS15 involved extracting genomic DNA, amplifying and sequencing its ribosomal internally transcribed spacer regions (ITS), and then applying an improved bioinformatics analysis based on existing methods. The detection of flanking ribosomal genes in the BS15 sequence by ITSx allowed for the extraction of full-length ITS1 and ITS2 sub-sequences, a critical factor for producing alignments where gap site data is utilized in the phylogenetic analyses [48]. Independent BLAST searches using these sub-sequences to query the UNITE+INSDC database returned alignments with species exclusively of the taxonomic family *Xylariaceae*. Therefore, all public sequence records pertaining to the family *Xylaraiaceae* were comprehensively screened. The ITS sequences were detected by ITSx in 3443 of 3470 records from 394 unique binomial species.

**Figure 2:**
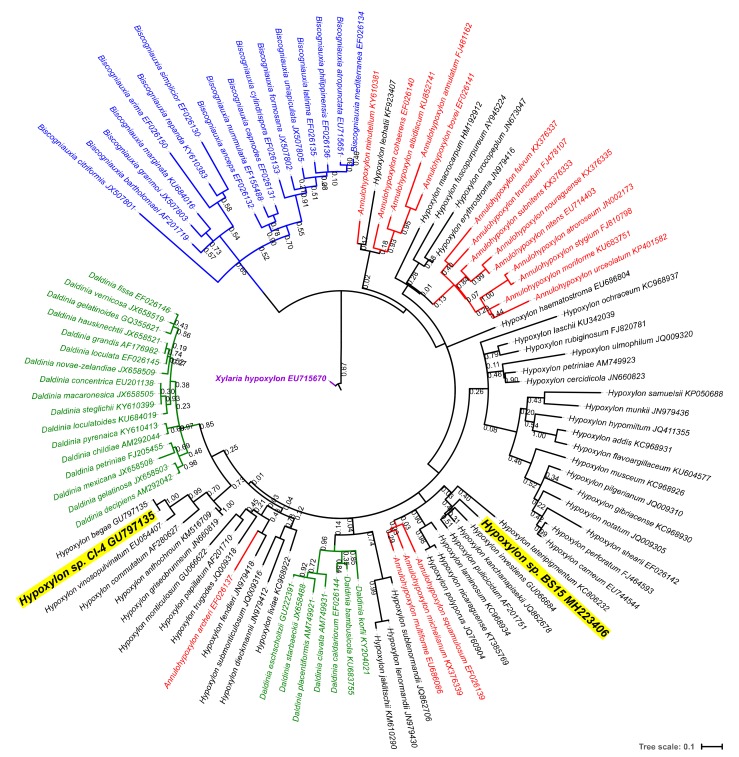
Phylogenetic reconstruction of *Hypoxylon* sp. BS15 and related organisms generated from maximum likelihood clustering of MUSCLE-aligned ITS2 sequences. Branch lengths are drawn to scale, representing the average number of nucleotide substitutions per site between the sequences. 121 nodes were selected for inclusion in the present figure from 331 nodes in the original phylogram. The bootstrap values at the nodes are from 1000 bootstrap iterations.

A notable discrepancy regarding the naming and classification of organisms described in this work is the recent recognition of the family *Hyopxylaceae* by INSDC, whose members were previously included within *Xylariaceae* [49]. However, these records have not yet been updated in UNITE at this time. For the present work, non-*Hypoxylaceae* species were included in the alignment and clustering but pruned from the ITS phylogram with the exception of *Xylaria hypoxylon,* presented as a rooted out-group (Figure 2).

The relative richness of the full-length sequence records and the consistency in the sequence length made ITS2 a more favorable target for multiple alignment than ITS1 for the family *Xylariaceae*. An analysis of ITSx outputs revealed a bias for sequences containing the large ribosomal subunit sequence (LSU) compared with the small subunit (SSU) sequences among the UNITE records for the family *Xylariaceae*. Because the detection of these flanking ribosomal sequences is required for the full-length extraction of ITS sequences by ITSx, there were nearly twice the number of full-length ITS2 sequences (*n* = 2165) available for alignment compared with ITS1 (*n* = 1212). The sequence lengths were considerably less variable for ITS2 (SD = 5) than ITS1 (SD = 51).

**Figure 3:**
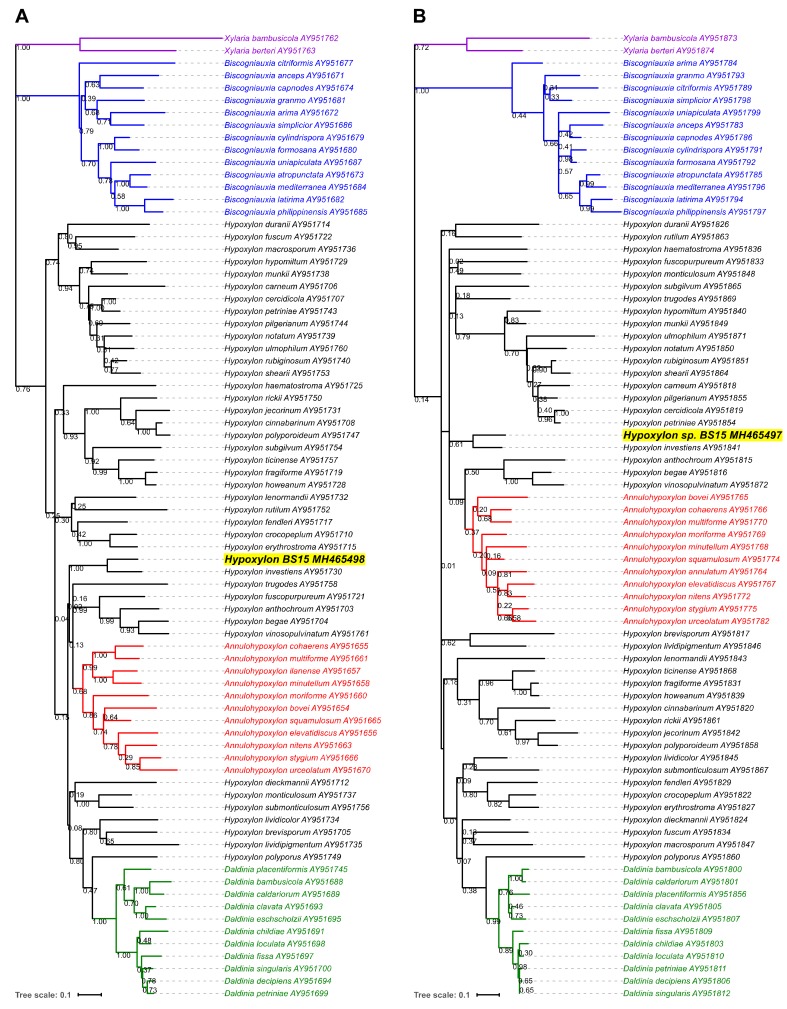
Phylogenetic reconstruction of *Hypoxylon* sp. BS15 and related organisms generated from maximum likelihood clustering of MUSCLE-aligned protein-coding gene sequences. A total of 78 sequences were analyzed for α-actin (**A**) and β-tubulin (**B**). The branch lengths are shown to scale, representing the average number of nucleotide substitutions per site between sequences. The bootstrap values at the nodes are from 1000 bootstrap iterations.

Our taxonomic evaluations are consistent with other authors who found protein-coding genes more congruent with phenotypic observations than non-coding ITS sequences for *Hypoxylon* and related genera [41]. The phylograms generated from the ITS2 sequences were remarkably unresolved regardless of the alignment and clustering methods, with several genera not clustered into the monophyletic groups (e.g., *Annulohypoxylon* spp., *Daldinia* spp.) (Figure 2). Both the α-actin and β-tubulin genetic analyses were able to fully resolve these taxa, albeit with fewer specimens (*n* = 78) than ITS (Figure 3). For all three genetic markers, the fungal strain BS15 was consistently clustered among *Hypoxylon* spp. and most closely associated with *H. investiens.*

### 3.3. Growth of BS15 on Degraded Cellulose and Analysis of Volatile Hydrocarbons

**Figure 4:**
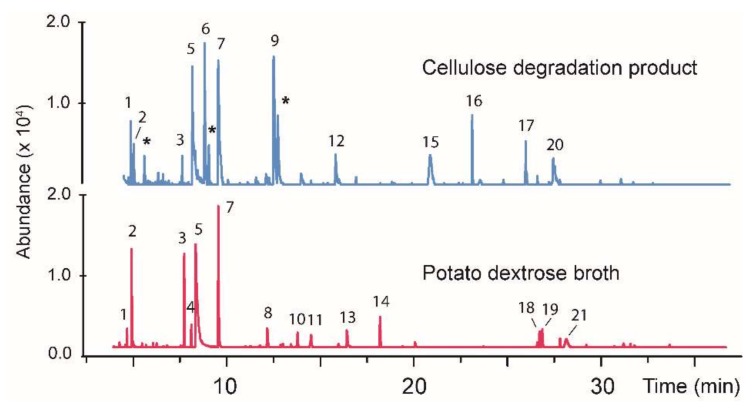
A gas chromatogram showing the volatile organic products produced by the fungus BS15 growth on a conventional media (**bottom**) and on carbohydrates produced from the degraded cellulose (**top**). The asterisks (*) denote volatile contaminants occurring in the degraded cellulose media as determined by analyzing a control sample with no BS15 added. The tentative identification and nominal masses of the individual peaks numbered in the chromatogram are given in Table 2.

**Figure 5:**
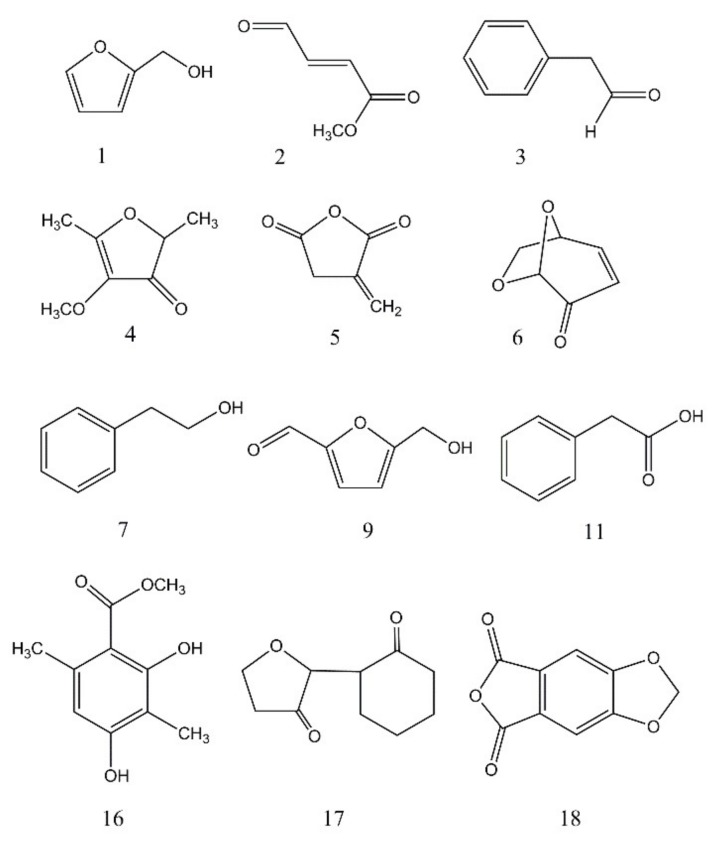
Structures of the volatile compounds tentatively identified from BS15 by comparison with the mass spectra in the NIST database. The compound numbers correspond to the peak numbers listed in Table 2 and in Figure 4.

In order to more generally evaluate the suitability of the degraded cellulose as a carbon source for fungi, BS15 was also evaluated for its ability to grow on the material. The procedure described above using two separate diets was employed with BS15 growth. The first included the degraded cellulose as the sole carbon source (see experimental), and the second contained potato dextrose broth. Both cultures exhibited strong fungal growth with mycelium covering the entire surface of the liquid media in approximately two weeks. The hydrocarbon fraction was extracted after three weeks using the solid-phase extraction process described above. A GC/MS analysis exhibited a large number of volatile products. A chromatographic comparison of the volatile compounds produced from each growth condition is illustrated in Figure 4, with tentative structural assignments and molecular weights listed in Table 2. The structures of the compounds listed in Table 2 are illustrated in Figure 5. In the case of BS15, both diets produced compounds **1**, **2**, **3**, **5**, and **7** but all the other products differed depending on the diet employed. Another notable difference in comparison with CI-4 is that BS15 on the degraded cellulose diet produced approximately the same amounts of volatile products as the potato dextrose diet.

In order to verify that the volatile compounds resulting from growth of BS15 on the degraded cellulose were being generated from simple sugars rather than from residual cellulose, a control was prepared containing non-degraded cellulose as described above for CI-4. After two weeks of growth on this cellulose medium, no growth was observed.

### 3.4. Comparing Solid-Phase and Liquid–Liquid Extraction Methods

**Figure 6:**
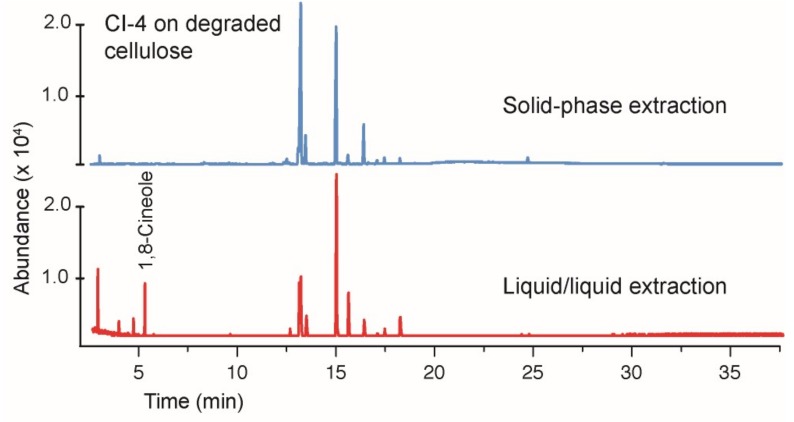
A comparison of the solid-phase extraction versus the liquid–liquid extraction (ethyl acetate/water). The growth media contained the degraded cellulose as a carbon source. A notable difference is the presence of 1,8-cineole in the liquid–liquid extraction. A comparison of the potato dextrose broth gave very similar results and, therefore, is not shown. The identity of 1,8-cineole was verified by comparison to an authentic standard.

All extractions of the volatile compounds in this study were performed using a solid-phase extraction with a C-18 stationary phase. This method is widely viewed as a “green chemistry” alternative to liquid–liquid extractions that require two–three orders of magnitude less solvent. However, it comes with the risk of potentially extracting fewer or different compounds from the growth media. In order to verify that the solid-phase extraction was effectively extracting the growth media, a direct comparison was made versus a liquid–liquid extraction using ethyl acetate/water. The comparisons were made for CI-4 and for BS15. For CI-4, Figure 6 illustrates that the solid-phase extraction gave very similar results, while using approximately 100 times less solvent. The differences in the relative amounts of certain products are notable. For example, the liquid–liquid extraction includes 1,8-cineole, while this product is missing from the solid-phase extraction. At present, the cause of this difference is unknown and further study is needed. Overall, however, the majority of the compounds extracted are the same using either method, and each technique extracts similar amounts as judged by the similar peak areas for the signals having the same retention times.

**Figure 7:**
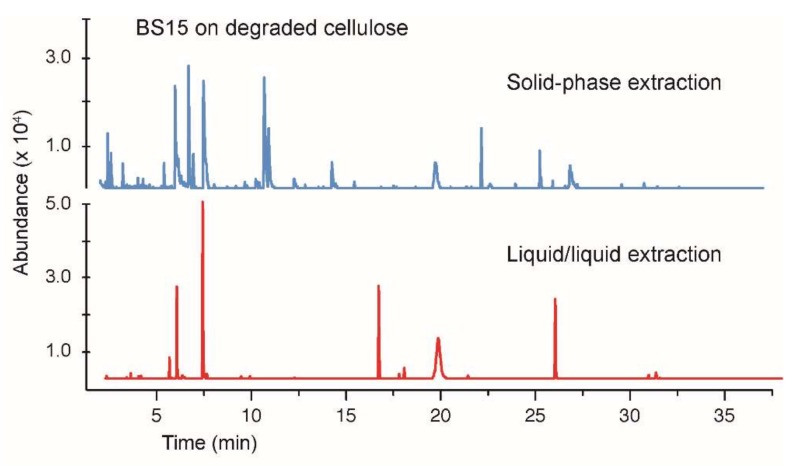
A comparison of the solid-phase extraction versus the liquid–liquid extraction (ethyl acetate/water). The growth media contained BS15 grown on the degraded cellulose as a carbon source.

A similar comparison of the effectiveness of the liquid–liquid extraction versus the solid-phase extraction was performed using BS15 grown on the degraded cellulose (Figure 7). Here, the differences between the methods were more pronounced with the solid-phase process extracting a more diverse range of products than did the liquid–liquid extraction. The absence of cineole in Figure 7 is a notable omission.

The solid-phase extraction process used herein differs from the vast majority of prior studies on volatile products from fungi, which have utilized solid-phase micro-extraction (SPME). This choice was made because SPME preferentially measures compounds having significant populations in the vapor phase and thus biases analysis against materials having low vapor pressures. Because compounds having potential use as fuels may have low vapor pressure, a methodology was employed here that includes these compounds. Admittedly, the use of a solid-phase extraction (SPE) represents a significant deviation from the common practice in the analysis of volatiles, and future work is needed to directly compare SPE and SPME to clearly identify the advantages and limitations.

## 4. Conclusions

The work described herein establishes the ability of certain endophytic fungi to convert a mechanochemically degraded cellulose product into volatile organic products with potential relevance as fuels. This process has been shown to be feasible with two different *Hypoxylon* sp. to demonstrate that the results are not limited to a single organism. One of the fungi employed (BS15) is described here for the first time, and the phylogenetic analysis demonstrated that it is substantially divergent from any other named species. BS15 produced a range of volatile products that differed significantly from those previously described from CI-4, emphasizing the importance of intra-generic species variation in metabolic studies. Here, the measurement of the total concentration of volatile products is not reported, because the quantification of the individual peaks in the chromatograms is not possible without standards and, at present, several products remain unknown. Our future work will focus on a more complete characterization of the individual compounds and the measurement of the total production.

## Figures and Tables

**Table 1:** Waste materials containing cellulose that can be converted into simple sugars.

Feedstock	Percent Hydrolyzed *^a^*
Cherry pit	95.7
Flint corn kernals	93.4
St. Augustine grass	92.5
Oat	90.3
Orange peel	85.0
Corn cobs	81.5
Bamboo	75.1
Cedar	74.0
Red Oak	72.4
Maple	72.0
Douglas Fir	71.1
*Nannochloropsis*	69.2
Aspen	68.0
Poplar	66.9
Yellow pine	65.3
Wheat	65.0
*Miscanthus* grass	64.7
White pine	64.4
Mixed yard waste	58.1
Switch grass	57.9
Hickory	55.9
Paper, newsprint	54.7
Flint corn stover	52.1
Banana leaf	52.0
Big blue stem grass	50.1
Little blue stem grass	48.9
Coffee grounds	45.2

*^a^* This value represents hydrolysis of the holocellulose present in the material.

**Table 2:** A list of the volatile compounds produced by CI-4 or BS15 grown on either the potato dextrose broth (PD) or the degraded cellulose (DC), showing tentative compound identification where possible.

Fungus	Peak # *^a^*	R.T. (min)	Area (%) PD, DC *^b^*	Tentative Identity *^c^*	Mol. mass (Da)	Qual. *^d^*
CI-4	1	9.58	0.1, -	Unknown	126	-
CI-4	2	9.72	0.3, -	Unknown	138	-
CI-4	3	10.88	0.5, -	Unknown	124	-
CI-4	4	12.93	0.2, -	Unknown	152	-
CI-4	5	13.43	0.8, -	Unknown	122	-
CI-4	6	13.57	0.3, -	Unknown	154	-
CI-4	7	13.68	1.4, -	3-Ethenyl-2-methylene cyclopentanecarboxylic acid	152	50
CI-4	8	14.11	0.6, 2.8	Unknown	152	-
CI-4	9	14.25	10.6, 45.7	Unknown	150	-
CI-4	10	14.57	15.5, 6.0	Unknown	154	-
CI-4	11	15.08	0.3, -	Unknown	154	-
CI-4	12	15.44	0.3, -	Unknown	152	-
CI-4	13	16.02	43.8, 2.0	Unknown	168	-
CI-4	14	16.45	1.1, 2.0	1-Acetyl-2-(1-hydroxyethyl)-cyclohexene	168	50
CI-4	15	17.24	10.4, 7.6	Unknown	150	-
CI-4	16	17.79	1.0, 0.8	Unknown	170	-
CI-4	17	18.14	1.5, 1.2	Unknown	170	-
CI-4	18	18.84	1.1, 1.1	3-Isopropoxy 5-methyl-phenol	166	61
CI-4	19	24.78	4.0, 1.0	2,3-Dimethoxy-naphthalene	188	85
BS15	1	4.82	1.1, 2.7	Furfuryl alcohol	98	72
BS15	2	5.07	9.9, 2.4	Methyl 4-oxo-2-butenoate	114	94
BS15	3	7.85	9.3, 1.7	Benzeneacetaldehyde	120	70
BS15	4	8.22	1.6, -	4-methoxy-2,5-dimethyl-3 (2H)-furanone	142	77
BS15	5	8.45	36.7, 26.1	2,5-furandione dihydro-3-methylene	112	55
BS15	6	8.65	-, 21.5	Levoglucosenone	126	78
BS15	7	9.65	20.9, 12.8	2-Phenyethanol	122	86
BS15	8	12.24	3.0, -	Unknown	158	-
BS15	9	12.34	-, 20.3	5-(Hydroxymethyl)furfural	126	91
BS15	10	13.84	17.8	Unknown	86	–
BS15	11	14.56	2.5, -	Phenylacetic acid	136	75
BS15	12	16.23	3.0, -	Unknown	138	-
BS15	13	16.45	-, 3.2	Unknown	142	-
BS15	14	18.20	3.7, –	Unknown	154	-
BS15	15	20.47	-, 8.1	Unknown	162	-
BS15	16	22.73	-, 3.2	2,4-dihydroxy-3,6-dimethyl Benzoic acid, methyl ester	196	72
BS15	17	25.58	-, 2.1	Dihydro-5-(2-oxocyclohexylidene) 2(3H)-furanone	180	70
BS15	18	26.63	1.7, -	Furo [3, 4-f][1,3] benzodioxole-5,7-dione	192	65
BS15	19	26.78	2.9, -	Unknown	97	-
BS15	20	27.06	8.7, -	Unknown	127	-
BS15	21	28.04	1.8, -	Unknown	127	-

*^a^* Peak numbers correspond to the numbering shown in Figure 1 (CI-4) or Figure 4 (BS15). *^b^* The labels PD and DC refer, respectively, to the potato dextrose broth and the degraded cellulose. The areas listed are the relative peak areas. *^c^* All the assignments of structure were made on the basis of the match to the National Institute of Standards and Technology (NIST) database. *^d^* Qual. refers to the highest listed quality value for the peaks that occur in both the growth media ort, for the peaks that occur only in a single medium, to the value from that solution.
